# Editorial: Cytokines as Players of Neuronal Plasticity and Sensitivity to Environment in Healthy and Pathological Brain

**DOI:** 10.3389/fncel.2015.00508

**Published:** 2016-01-12

**Authors:** Silvia Alboni, Laura Maggi

**Affiliations:** ^1^Department of Life Sciences, University of Modena and Reggio EmiliaModena, Italy; ^2^Department of Physiology and Pharmacology, Sapienza UniversityRome, Italy

**Keywords:** cytokine, chemokines, microglia, neurons, astrocytes, neuronal plasticity, environment, behavior

In this e-Book, we collected recent evidence on the role of cytokines (including chemokines) in the interplay between environmental stimulation and central responses in both physiological and pathological conditions. The e-Book includes original studies and review articles focused on cytokine function during brain development as well as in the mature brain.

Cytokines, together with neurotransmitters and hormones, are signaling molecules playing a key role in the maintenance of neuro-immune-endocrine system homeostasis. The cytokine systems (constituted by cytokines, their receptors and regulators of their activity) are expressed throughout the brain and their expression is regulated during brain development until aging. Brain cells, including neurons as well as glia cells, can release and/or be responsive to cytokines, therefore these molecules can enable communication between different cell types. Under physiological conditions, cytokines typically participate in brain development and plasticity by translating environmental into molecular signals. However, once the allostatic equilibrium is compromised, cytokine systems, if over- or chronically-activated, may participate in mediating toxic effects in the brain. Indeed, a central role for cytokines in neuropsychiatric as well as neurodegenerative disorders is now well recognized.

The major source of cytokines release in the brain is microglia cells that are actively involved in adult brain homeostasis and in neural loss and synaptic maturation during development. The contribution of Pagani et al. addresses the role of fractalkine (CX3CL1) signaling in the developmental profile of morphological features and physiological properties of microglia using mice lacking the fractalkine receptor (who is expresses only in microglia within the healthy brain). Sheridan et al. investigated the role for fractalkine in synaptic plasticity showing that the levels of hippocampal fractalkine increases after a memory task and that the chemokine regulates glutamate-mediated neurotransmission tone. A comprehensive review on the role of fractalkine in regulating microglia properties, brain plasticity and behavior is provided by Paolicelli et al.. Another cytokine known to modulate memory-related processes is interferon (IFN)-γ. One of the article included in this e-book describe the effects induced by the lack of IFN-γ on memory function under basal or stressful conditions (Litteljohn et al.). This study emphasizes the importance of considering the “brain state” (healthy or disease) when the modulation of neurobehavioral processes by the cytokine systems it is evaluated. Interestingly, in the commentary of von Bohlen and Halbach, the astrocytic secreted lipocalin 2 is described as a mediator of the dialogue between astrocytes and neuron that in turn affects synaptic plasticity.

Neuroimmune factors are particularly relevant to a number of neurophatologies. They play important pro-survival and/or pro-death roles by regulating targets in specific brain regions. Sutinem et al. reported that in pathological conditions, such as Alzheimer's disease, the cytokine interleukin (IL)-18 seems potentially involved in driving protein changes relevant for the pathogenesis, whereas Chiavegato et al. described the role of IL-1 beta and the high mobility group B1 (HMGB1) in preventing seizure-like discharges in models of focal epilepsy.

Cytokines affect brain functions through different molecular mechanisms. Groul et al. show that up-regualtion of astrocytic CCL2 and Il-6 differentially affect the levels of specific proteins in cerebellum and hippocampus. The review of Guyon described how the stimulation of the receptor CXCR4 by the chemokine CXCL12 regulates the synaptic release of glutamate and γ-aminobutyric acid (GABA). Calabrese et al. proposed the neurotrophin brain-derived neurotrophic factor (BDNF) as a bridge between increased levels of pro-inflammatory cytokines and impaired neuroplasticity while the contribution of Cattaneo et al. is centered on epigenetics mechanisms that, by altering inflammation-immune systems and neuronal plasticity, may increase vulnerability to develop psychiatric disorders following early life stressful events.

Finally, in their review, Singhal et al. analyze the neuroimmune mechanisms associated with neurobiological and behavioral changes following different environmental stimulations. In particular, they described how environmental enrichment (mainly physical exercise) affects neuroimmune targets and behavior.

Overall, the work presented herein provides an insight into important aspects of the role of neuroimmune factors on brain activity and behavior. It represent a small windows on a complex panorama that still remain to be fully elucidated, especially with regard to the interplay between cytokine action, environmental stimulation and neuronal outcome.

## Conflict of interest statement

The authors declare that the research was conducted in the absence of any commercial or financial relationships that could be construed as a potential conflict of interest.

